# Inter-Species Differences in Regulation of the Progranulin–Sortilin Axis in TDP-43 Cell Models of Neurodegeneration

**DOI:** 10.3390/ijms20235866

**Published:** 2019-11-22

**Authors:** Valentina Gumina, Elisa Onesto, Claudia Colombrita, AnnaMaria Maraschi, Vincenzo Silani, Antonia Ratti

**Affiliations:** 1 Istituto Auxologico Italiano, IRCCS, Department of Neurology-Stroke Unit and Laboratory of Neuroscience, Via Zucchi 18, 20095 Cusano Milanino, Milan, Italy; valegumina@gmail.com (V.G.); elisa.onesto.eo@axxam.com (E.O.); claudiacolombrita@hotmail.com (C.C.); a.maraschi@auxologico.it (A.M.); vincenzo@silani.com (V.S.); 2 Department of Pathophysiology and Transplantation, “Dino Ferrari” Center, Università degli Studi di Milano, Via F. Sforza 35, 20122 Milan, Italy; 3 “Aldo Ravelli” Center for Neurotechnology and Experimental Brain Therapeutics, Università degli Studi di Milano, Via A. di Rudinì 8, 20142 Milan, Italy; 4 Department of Medical Biotechnology and Translational Medicine, Università degli Studi di Milano, Via Fratelli Cervi 93, 20090 Segrate, Milan, Italy

**Keywords:** progranulin, sortilin, TDP-43, ALS, FTLD

## Abstract

Cytoplasmic aggregates and nuclear depletion of the ubiquitous RNA-binding protein TDP-43 have been described in the autoptic brain tissues of amyotrophic lateral sclerosis (ALS) and frontotemporal dementia (FTLD) patients and both TDP-43 loss-of-function and gain-of-function mechanisms seem to contribute to the neurodegenerative process. Among the wide array of RNA targets, TDP-43 regulates progranulin (*GRN*) mRNA stability and sortilin (*SORT1*) splicing. Progranulin is a secreted neurotrophic and neuro-immunomodulatory factor whose endocytosis and delivery to the lysosomes are regulated by the neuronal receptor sortilin. Moreover, *GRN* loss-of-function mutations are causative of a subset of FTLD cases showing TDP-43 pathological aggregates. Here we show that TDP-43 loss-of-function differently affects the progranulin–sortilin axis in murine and human neuronal cell models. We demonstrated that although TDP-43 binding to *GRN* mRNA occurs similarly in human and murine cells, upon TDP-43 depletion, a different control of sortilin splicing and protein content may determine changes in extracellular progranulin uptake that account for increased or unchanged secreted protein in murine and human cells, respectively. As targeting the progranulin–sortilin axis has been proposed as a therapeutic approach for *GRN*-FTLD patients, the inter-species differences in TDP-43-mediated regulation of this pathway must be considered when translating studies from animal models to patients.

## 1. Introduction

The ubiquitous TDP-43 RNA-binding protein (RBP) is the major component of the pathological aggregates described in the autoptic brain tissues of the majority of amyotrophic lateral sclerosis (ALS) patients and of a subset of frontotemporal lobar dementia (FTLD) cases [1,2]. Pathological TDP-43 aggregates are prevalently localized in the cytoplasm of affected neurons, which typically also show a concomitant depletion of TDP-43 protein from the nucleus [1,2]. The pathobiology associated to TDP-43 therefore includes both gain- (GOF) and loss-of-function (LOF) mechanisms and a combination of both is supposed to contribute to the neurodegenerative process in ALS/FTLD diseases [3].

TDP-43 regulates different steps of RNA metabolism both in the nucleus, where it is mainly localized and controls splicing, and in the cytoplasm, where it is involved in mRNA transport, stability and local translation [4]. A plethora of TDP-43 splicing and mRNA targets have been identified by high-throughput approaches in physiological and disease states [5]. We and others found progranulin (*Grn*) mRNA as a cytoplasmic target in murine experimental models with TDP-43 binding to its 3′UTR sequence [6,7]. We also demonstrated that, upon TDP-43 LOF in motoneuronal-like NSC-34 cells, *Grn* mRNA stability is increased, leading to an upregulation of progranulin (Pgrn) protein content [7].

Progranulin is a secreted glycoprotein expressed in the nervous system both in neuronal and glial cells, where it mainly modulates neuroinflammation and acts as a neurotrophic factor, promoting cell survival and neurite/axon growth [8]. Autosomal dominant LOF mutations in the *GRN* gene are causative of familial (5–20%) and sporadic (1–5%) FTLD cases [9], which also present with TDP-43 pathological aggregates in brain tissues [10], suggesting a mechanistic link between *GRN* haploinsufficiency and TDP-43 pathology. Both in animal and cell models, Pgrn depletion was shown to induce cytoplasmic TDP-43 mislocalization or accumulation of its C-terminal fragments and to severely compromise neuronal survival and neurite formation [11,12,13,14].

The maintenance of progranulin levels is also important for lysosome activity, which is severely affected in *Grn* knock-out mice [15] and in neuronal ceroid lipofuscinosis (NCL), a disease due to rare recessive *GRN* LOF mutations [16,17]. In neurons, progranulin homeostasis and delivery to lysosomes is regulated by its interaction with the transmembrane receptor sortilin (SORT1) [18], recently identified as a rare genetic risk factor for FTLD [19]. Interestingly, TDP-43 also regulates *SORT1* gene expression [20] and alternative splicing, although producing different isoforms in mice and in humans [6,21,22]. In particular, TDP-43 represses the inclusion of an intronic exon cassette (exon 17b) which, in the case of TDP-43 LOF, generates a longer Sort1 protein with a function similar to the main Sort1 isoform lacking the 33-aminoacidic region encoded by exon 17b (Sort1∆ex17b) in mice [21]. In contrast, in humans, the inclusion of this exon cassette, although a rarer event than in mice, introduces a premature stop codon leading to a non-functional and extracellularly released SORT1 protein that may act as a decoy receptor, inhibiting PGRN endocytosis [21,22].

Therapeutic approaches for FTLD-*GRN* pathology aim to restore PGRN levels by the inhibition of the SORT1–PGRN interaction. Indeed, the pharmacological or gene inhibition of SORT1 protein levels has been associated with an increase of extracellular PGRN levels [18,23]. Moreover, PGRN treatment or overexpression exerts a neuroprotective effect on cultured neurons [24] and is able to rescue neuronal defects and TDP-43 aggregation both in zebrafish and mice models of TDP-43 pathology [25,26,27].

Given the TDP-43 regulatory activity on both *GRN* and *SORT1* RNA, in this study we further investigated the progranulin–sortilin axis in TDP-43 LOF cell models, evaluating if the secreted progranulin levels, important for both its neurotrophic and lysosomal functions, are affected. By comparing human and murine TDP-43 LOF neuronal cell models, we provide evidence that TDP-43-associated regulatory mechanisms differ between mice and humans having a different impact on progranulin bioavailability.

## 2. Results

### 2.1. Analysis of Intracellular and Secreted Pgrn Protein in Murine TDP-43 LOF and GOF Cell Models 

**Figure 1:**
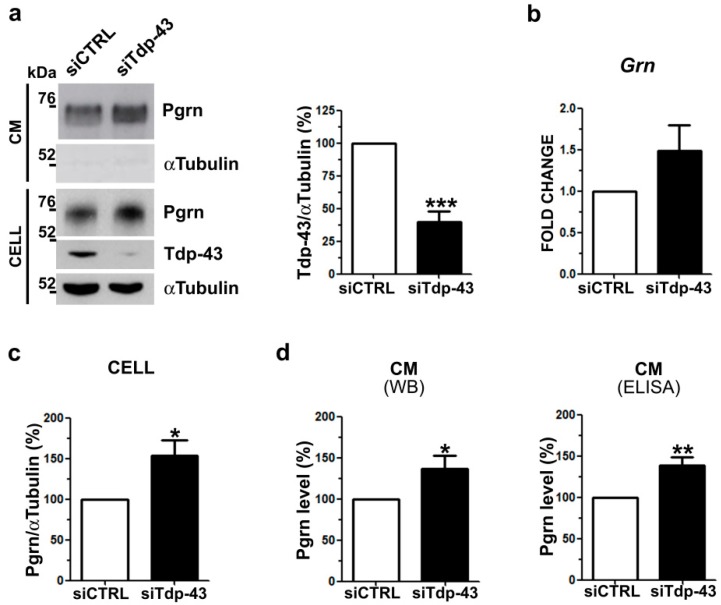
Progranulin (Pgrn) protein content and secretion in murine TDP-43 loss-of-function (LOF) cells. (**a**) Representative Western blot (WB) images (left panel) of Pgrn protein in cell lysates (CELL) and conditioned media (CM) upon Tdp-43 knock-down (siTdp-43) in murine motoneuronal-like NSC-34 cells compared to negative control siRNA-transfected (siCTRL) cells. Densitometric analysis (right panel) shows Tdp-43 gene silencing efficiency. αTUBULIN was used for intracellular data normalization and as a non-secreted control protein in CM (mean ± s.e.m.; *n* = 5 independent experiments; two-tailed unpaired *t* test; *** *p* < 0.001). (**b**) Real time PCR of *Grn* gene expression upon Tdp-43 depletion (mean ± s.e.m.; *n* = 3 independent experiments; two-tailed unpaired *t* test). (**c**) Densitometric and statistical analysis of intracellular (CELL) Pgrn protein content shown in (**a**) (mean ± s.e.m.; *n* = 5 independent experiments; two-tailed unpaired *t* test; * *p* < 0.05). (**d**) Secreted Pgrn levels detected in CM by WB shown in (**a**) and by ELISA assays (mean ± s.e.m.; *n* = 4 independent experiments; two-tailed unpaired *t* test; ** *p* < 0.01).

We previously demonstrated that intracellular Pgrn levels are up-regulated by Tdp-43 LOF in murine motoneuronal-like NSC-34 cells [7]. As extracellular progranulin is important to exert its physiological functions in the nervous system, we investigated if Tdp-43 depletion also affects secreted Pgrn levels. Upon Tdp-43 knock-down in NSC-34 cells (Figure 1a), we confirmed a significant 1.5-fold increase of Pgrn protein content in cell lysates (Figure 1a,c) and a similar, although not significant, trend for *Grn* gene expression (Figure 1b), as previously reported [7]. When we measured Pgrn content in the conditioned media by Western blot (WB) and ELISA assays, we observed a significant and similar 1.4-fold increase in secreted Pgrn in Tdp-43-knocked-down cells compared to control cells (Figure 1a,d). To confirm that Tdp-43 depletion affects Pgrn protein content, we analyzed another murine neuronal Tdp-43 LOF cell model. Upon Tdp-43 knock-down in murine neuroblastoma N2a cells, a significant increase of both intracellular (2.1-fold) and secreted (1.6-fold) Pgrn protein levels were observed, although *Grn* mRNA levels remained unchanged (Supplementary Figure S1), confirming the results obtained in NSC-34 cells.

**Figure 2:**
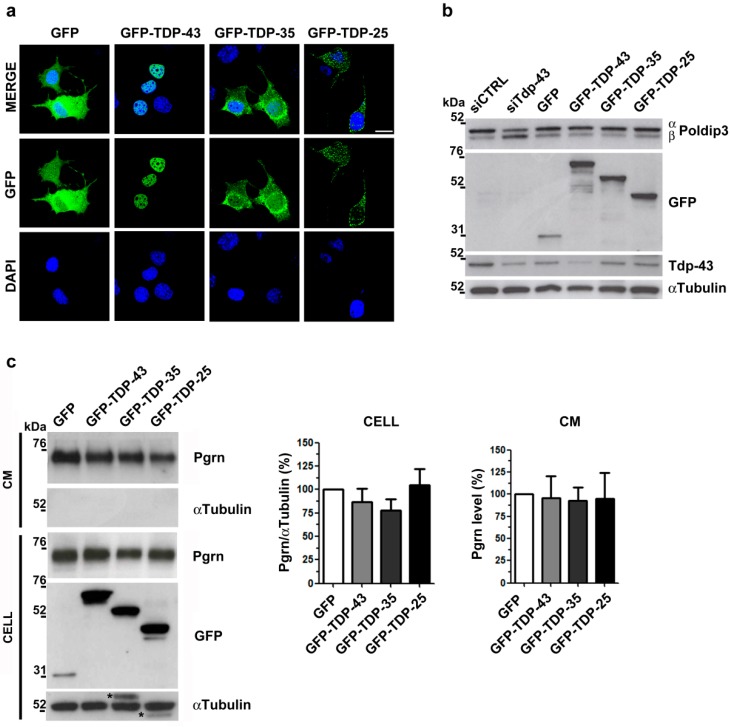
Pgrn protein content and secretion in murine TDP-43 gain-of-function (GOF) cells. (**a**) Representative immunofluorescence images showing the subcellular localization of the recombinant GFP-TDP-43 full length and C-terminal fragments (GFP-TDP-35; GFP-TDP-25) in NSC-34 cells. DAPI (blue) was used for nuclei staining. Scale bar: 10 µm. (**b**) Representative WB images of Poldip3 protein isoforms (α and β) in NSC-34 cells knocked-down for Tdp-43 (siTdp-43) or expressing GFP-TDP-43, GFP-TDP-35 and GFP-TDP-25 constructs as indicated. Immunoblots with anti-GFP and anti-Tdp-43 antibodies were performed to assess TDP-43 over-expression and gene silencing efficiencies, respectively. Upon GFP-TDP-43 over-expression, autoregulation of the endogenous Tdp-43 protein could be observed. αTubulin was used for sample normalization. (**c**) Representative WB images and densitometric analyses of Pgrn protein content in cell lysates (CELL) and conditioned media (CM) of NSC-34 cells transfected as indicated. αTubulin was used for sample normalization and as non-secreted control protein in CM (mean ± s.e.m.; *n* = 3 independent experiments; one-way ANOVA and Bonferroni’s multiple comparison post hoc test) *, residual signal from previous anti-GFP hybridization.

To assess if Tdp-43 affects Pgrn protein content specifically by a LOF mechanism, we also analyzed Pgrn protein levels in a TDP-43 gain-of-function (GOF) model obtained by the ectopic expression of the pathological TDP-43 C-terminal fragments (25 kDa and 35 kDa). Unlike the full-length TDP-43 protein, which localized only in the nucleus as expected, we confirmed that the truncated 35 kDa and 25 kDa TDP-43 proteins formed aggregates with a prevalent cytoplasmic localization (Figure 2a), mimicking the pathological aggregation observed in ALS/FTLD brains [28]. To further characterize these TDP-43 GOF models and thereby exclude a loss of the endogenous TDP-43 function, we analyzed the splicing pattern of the well-known TDP-43 target *POLDIP3* [29,30]. When the recombinant GFP-TDP-43, GFP-TDP-35 and GFP-TDP-25 proteins were over-expressed in NSC-34 cells, the main Poldip3 α protein isoform was expressed similarly to the control condition (GFP) (Figure 2b), suggesting that the endogenous Tdp-43 splicing activity is maintained in these TDP-43 GOF models in line with previous data [28]. In contrast, upon TDP-43 knock-down, we observed an increase of the Poldip3 β isoform compared to control cells (Figure 2b) as expected [29]. When we measured Pgrn protein by WB assay in TDP-43 GOF cells, we did not observe any difference in its level both in the cell lysates and the conditioned media (Figure 2c). These data suggest that both intracellular and secreted Pgrn protein levels are specifically affected by Tdp-43 LOF mechanism in murine neuronal NSC-34 and N2a cells.

### 2.2. Analysis of Intracellular and Secreted PGRN Protein in Human TDP-43 LOF and GOF Cell Models 

To further investigate if the TDP-43-mediated regulation of Pgrn protein content observed in mice is also conserved in humans, we first tested if TDP-43 is able to bind the human *GRN* mRNA as already proven in mice [7]. Alignment of the murine and human *GRN* 3′UTR sequences showed an overall 70% identity (Supplementary Figure S2). In the human sequence, a 24-nucleotide long region including a (TG)_5_ motif was predicted as the putative binding site for TDP-43 by in silico analysis with the RBPmap software compared to the highly conserved murine region embedded with a (TG)_6_ motif (Supplementary Figure S2).

**Figure 3:**
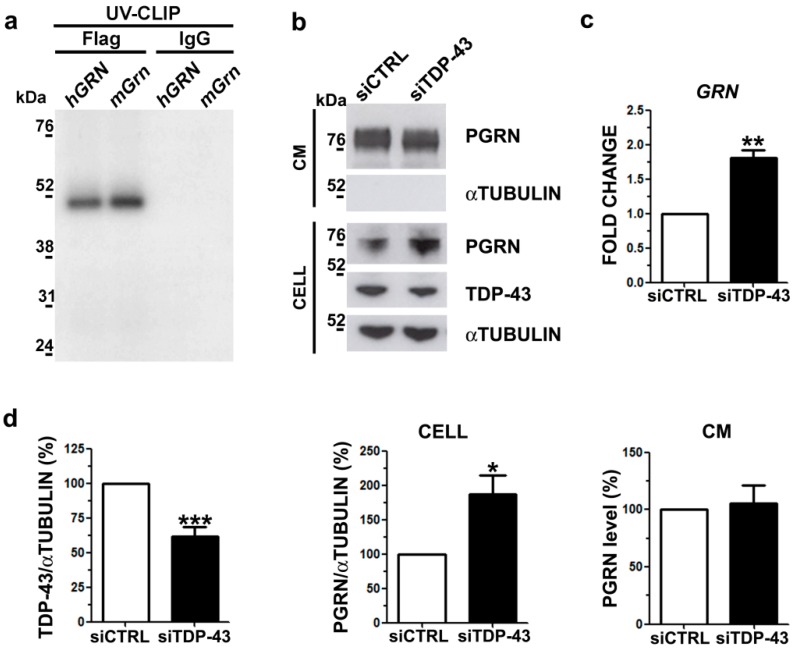
PGRN protein content and secretion in human TDP-43 LOF cells. (**a**) SDS-PAGE showing the results of the UV-crosslinking immunoprecipitation (UV-CLIP) experiment using HEK293T protein lysates overexpressing the human recombinant Flag-TDP-43 protein and the P^32^-radiolabelled murine and human *GRN* 3′UTR riboprobes. The murine riboprobe was used as positive control and the anti-IgG antibody as a negative control for immunoprecipitation. (**b**) Representative WB images showing TDP-43 and PGRN immunoblots of cellular lysates (CELL) and conditioned media (CM) in human M17 cells upon TDP-43 gene silencing. αTubulin was used for data normalization of intracellular protein content and as non-secreted control protein in CM. (**c**) Real time quantification of *GRN* mRNA upon TDP-43 knock-down in human neuroblastoma M17 cells (mean ± s.e.m.; *n* = 3 independent experiments; two-tailed unpaired *t* test; ** *p* < 0.01). (**d**) Densitometric and statistical analysis of WB data shown in **b**) (mean ± s.e.m.; *n* = 5 independent experiments; two-tailed unpaired *t* test; * *p* < 0.05; *** *p* < 0.001).

We performed a UV-crosslinking immunoprecipitation (UV-CLIP) assay using a riboprobe encompassing the human full-length *GRN* 3′UTR region (Supplementary Figure S2) and cell lysates expressing the human recombinant Flag-TDP-43 protein. Our UV-CLIP assay showed that the TDP-43 protein binds to the human *GRN* 3′UTR similarly to the murine one (Figure 3a), suggesting the conservation of post-transcriptional regulatory mechanisms between mice and humans on *GRN* mRNA.

To further investigate the regulatory activity of TDP-43 on PGRN in human cells, we analyzed the effects of TDP-43 LOF on cellular and secreted PGRN protein content in human neuroblastoma M17 cells. TDP-43 knock-down (Figure 3b,d) promoted a significant 1.8-fold increase of *GRN* mRNA levels (Figure 3c), also suggesting that human TDP-43 may act to reduce *GRN* mRNA stability as already demonstrated in mice [7]. When we evaluated changes at protein level, we observed a significant 1.9-fold increase of the intracellular PGRN levels (Figure 3b,d) in line with the mRNA data (Figure 3c) and similarly to Pgrn protein content in murine TDP-43 LOF cells (Figure 1). However, when we analyzed the secreted PGRN levels, we detected no changes upon TDP-43 knock-down (Figure 3b,d), in contrast with the murine LOF cell model (Figure 1).

**Figure 4:**
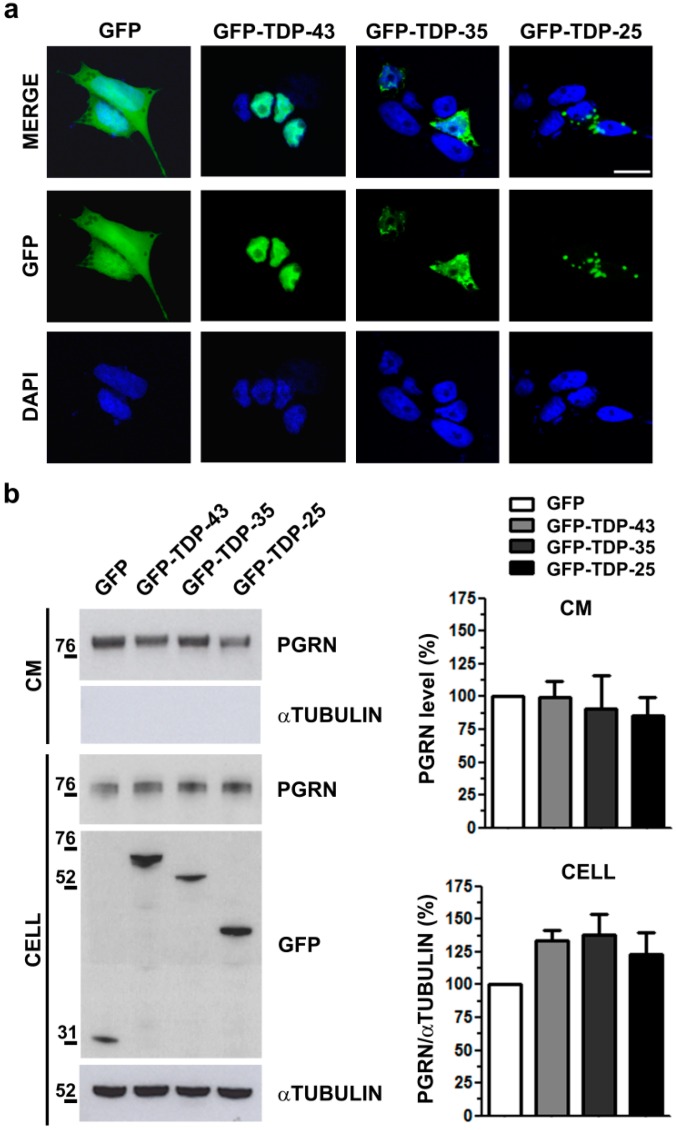
PGRN protein content and secretion in human TDP-43 GOF cells. (**a**) Representative immunofluorescence images showing the subcellular localization of the recombinant GFP-TDP-43 full-length and C-terminal fragments (GFP-TDP-35; GFP-TDP-25) in human neuroblastoma M17 cells. DAPI (blue) was used for nuclei staining. Scale bar: 10 µm. (**b**) Representative WB images and densitometric analyses of PGRN protein content in cell lysates (CELL) and conditioned media (CM) of M17 cells transfected as indicated. Anti-GFP immunoblot shows the transfection efficiency and αTubulin was used for sample normalization of the intracellular protein content and as non-secreted control protein in CM (mean ± s.e.m.; *n* = 3 independent experiments; one-way ANOVA and Bonferroni’s multiple comparison post hoc test).

To investigate if PGRN content is affected specifically by a TDP-43 LOF mechanism in humans similarly to mice, we reproduced a TDP-43 GOF model in human neuroblastoma M17 cells by the overexpression of the pathological TDP-43 C-terminal fragments (35 kDa and 25 kDa). By immunofluorescence analysis, we observed that the ectopic full-length TDP-43 protein localized in the nucleus and the 35 kDa and 25 kDa TDP-43 C-terminal fragments formed mainly cytoplasmic aggregates (Figure 4a) as in the murine NSC-34 cells (Figure 2a). WB analyses of intracellular and secreted PGRN protein levels did not show any difference in TDP-43 GOF models (Figure 4b), suggesting that also in human neuronal-like cells, PGRN regulation is impaired only by TDP-43 depletion, similarly to murine cells.

### 2.3. Analysis of Sortilin Splicing and Protein Levels upon TDP-43 LOF in Murine and Human Neuronal Cell Models

**Figure 5:**
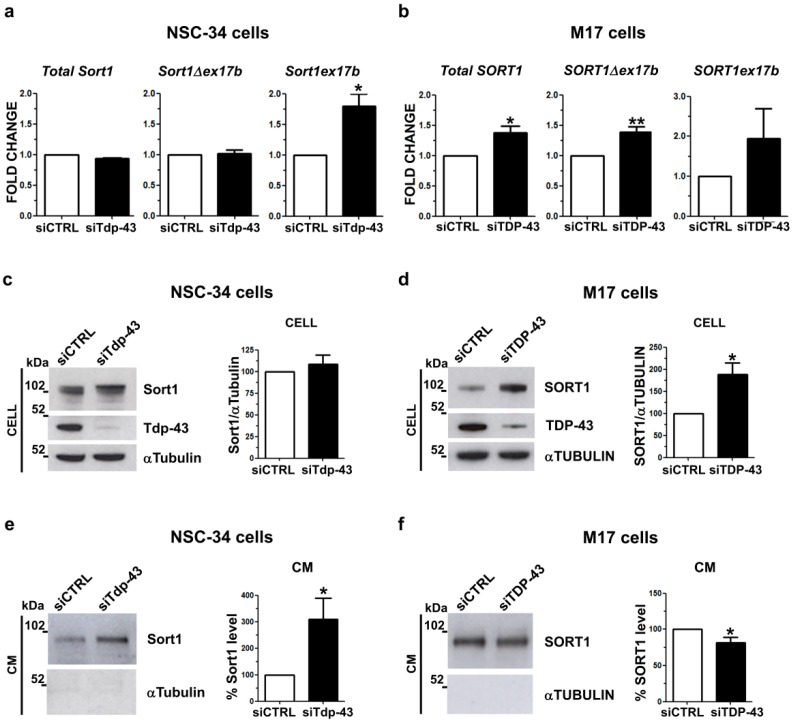
Regulation of sortilin gene expression, splicing and protein levels in human and murine TDP-43 LOF cells. Real time quantification of total *Sortilin* and of the splicing isoforms (∆*ex17b* and *ex17b*) transcripts upon TDP-43 depletion by siRNA in (**a**) NSC-34 cells and (**b**) M17 cells (mean ± s.e.m.; *n* = 3 independent experiments; two-tailed unpaired *t* test; * *p* < 0.05, ** *p* < 0.01). Representative WB and densitometry analyses of intracellular sortilin protein levels in (**c**) NSC-34 and (**d**) M17 cells. Immunoblots with TDP-43 antibody were shown to assess gene silencing efficiencies. αTubulin was used for sample normalization (mean ± s.e.m.; *n* = 5 independent experiments; two-tailed unpaired *t* test; * *p* < 0.05). WB and densitometric data of sortilin protein detected in the conditioned media (CM) of **e**) NSC-34 and **f**) M17 cells knocked-down for TDP-43. αTubulin was used as a negative control in CM (mean ± s.e.m.; *n* = 4 independent experiments; two-tailed unpaired *t* test; * *p* < 0.05).

As the secreted progranulin levels differ between the human and the murine TDP-43 LOF neuronal cell models, we hypothesized that these differences could be explained by a different progranulin uptake via its main neuronal transmembrane receptor, sortilin (SORT1), whose alternative splicing is described to be differently regulated by TDP-43 in mice and humans [21,31]. We analyzed sortilin gene expression and exon 17b alternative splicing by real time PCR in both murine motoneuronal-like NSC-34 and human neuroblastoma M17 cells knocked-down for TDP-43 (Figure 5). In murine NSC-34 cells, we observed that TDP-43 gene silencing induced a significant 1.8-fold increase of the *Sort1ex17b* isoform with unchanged overall expression of the *Sort1* gene (Figure 5a), similarly to what we observed in another murine neuronal-like cell model, the neuroblastoma N2A cells (Supplementary Figure S3), and confirming previous literature data [6,21]. In contrast, upon TDP-43 depletion in human neuroblastoma M17 cells, overall *SORT1* mRNA levels significantly increased 1.4 fold, as we previously described [20], mainly due to an increase of the main *SORT1Δex17b* isoform (Figure 5b) as the *SORT1ex17b* isoform represents a rare splicing isoform in human cells [21]. By analyzing SK-N-BE neuroblastoma cells as an alternative human neuronal cell model, we observed the same *SORT1* gene expression pattern of M17 cells (Supplementary Figure S4a).

When we analyzed Sort1 at the protein level in murine LOF cells, we observed that the total protein amount did not change in comparison with cells transfected with a control siRNA (Figure 5c, Supplementary Figure S3b). Instead, in human M17 cells, TDP-43 LOF led to a significant 1.9-fold increase in total SORT1 protein levels (Figure 5d), which was also confirmed in human neuroblastoma SK-N-BE cells (Supplementary Figure S4b).

As previous literature data showed that both the human and murine sortilin protein may be proteolytically cleaved and extracellularly released by the shedding mechanism [21,32], we also quantified soluble sortilin protein levels in the conditioned media by WB assay. In both murine and human cell models, the secreted sortilin protein showed a lower molecular weight (approximately 100 kDa) with respect to the intracellular sortilin protein (>100 kDa) (Figure 5e,f; Supplementary Figures S3c and S4c), corresponding to the soluble sortilin ectodomain after the proteolytic cleavage [21,32]. Upon Tdp-43 knock-down in murine NSC-34 cells, we observed a significant 3-fold increase in the secreted Sort1 protein (Figure 5e), supporting the hypothesis that the Sort1ex17b isoform, the one mainly expressed upon Tdp-43 knock-down, may be more accessible to protease cleavage and consequently more released extracellularly [21]. Similar results were obtained in murine N2a cells, where a 5-fold increase in Sort1 protein in the conditioned media was observed (Supplementary Figure S3c). However, in human M17 cells, the extracellular SORT1 protein level did not change upon TDP-43 depletion (Figure 5f), although its total intracellular content increased (Figure 5d). Similar results were obtained when we analyzed SORT1 in the conditioned media of human SK-N-BE cells (Supplementary Figure S4c). Altogether, our results show that sortilin receptor splicing and protein content is differently regulated by TDP-43 LOF in murine and human cell models and this may account for the observed inter-species differences in secreted progranulin levels.

## 3. Discussion

**Figure 6:**
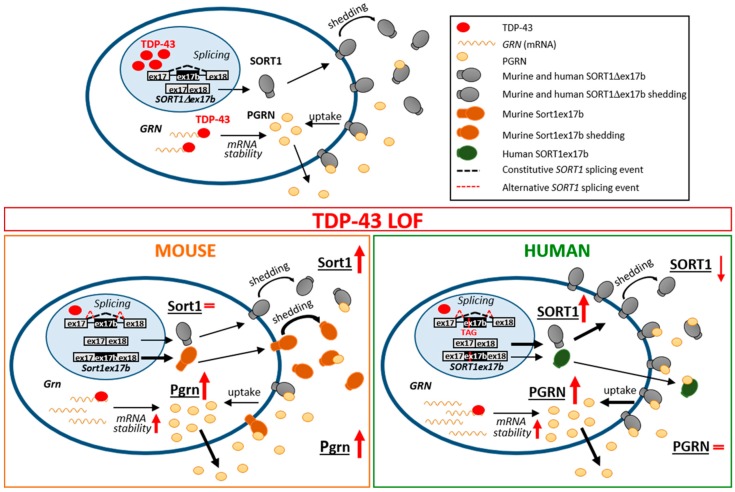
Proposed model for TDP-43 LOF effects on the PGRN–SORT1 axis in murine and human neuronal cells. In the nucleus, TDP-43 represses the inclusion of *SORT1ex17b*, an intronic exon cassette, while, in the cytoplasm, it binds to the *Progranulin* 3′UTR, decreasing its mRNA stability (upper panel). In the condition of TDP-43 LOF, intracellular progranulin protein increases in both cell models, while the secreted levels are upregulated in murine cells and unchanged in human ones (lower panels). We speculate that this difference is due to TDP-43-dependent regulation of gene expression and splicing of the *Sortilin* gene. Loss of TDP-43 promotes the inclusion of *SORT1ex17b*. While this alternative splicing event becomes prevalent in mice with the synthesis of a functional isoform more prone to shedding and to be released (left panel), it is rare in humans where the resulting isoform is instead truncated and not anchored to the membrane [21] (right panel). Moreover, in human cells, the observed increase of intracellular SORT1 protein (soluble and transmembrane) is likely to favor PGRN uptake, thus accounting for the differences in progranulin bioavailablity between mice and humans. Arrow thickness indicates upregulated or downregulated events compared to the physiological condition in the upper panel; red arrows indicate TDP-43 LOF effects.

In this study we found that TDP-43 LOF differently affects the progranulin–sortilin axis in murine and human cell models, which has an impact on secreted progranulin levels. In particular, we showed that in both murine and human neuronal-like cells the intracellular and extracellular progranulin protein levels are affected specifically by a TDP-43 LOF, while the presence of TDP-43 cytoplasmic aggregates seems not to influence progranulin homeostasis. In particular, we observed an increase of the intracellular progranulin protein levels both in murine and human TDP-43 knocked-down cells, while the secreted progranulin content was upregulated in the two murine cell models and was unchanged in the human ones. Our data show that the inter-species differences in progranulin bioavailability may be explained by a different TDP-43-dependent regulation of gene expression and splicing of the *Sortilin* gene and by the subsequent different distribution of the sortilin receptor both intracellularly and extracellularly (Figure 6).

We previously described that TDP-43 post-transcriptionally regulates the *Grn* gene by binding to its 3′UTR sequence and promoting its mRNA instability in murine NSC-34 cells [7], confirming previous data that identified *Grn* as an in vivo RNA target of TDP-43 in the adult mouse brain, where TDP-43 depletion causes an increase in *Grn* mRNA [6]. Given the high conservation between murine and human 3′UTR sequences, here we showed that TDP-43 binding to human *GRN* mRNA is conserved and that TDP-43 depletion upregulates *GRN* gene expression, suggesting that TDP-43-mediated regulation of *GRN* mRNA instability is also likely to be maintained in humans. Indeed, recent findings have demonstrated that TDP-43 promotes *GRN* mRNA instability by acting on the deadenylation process in human cell models [33]. In rodent and human models, TDP-43 is known to regulate the stability of other mRNAs, with either destabilizing (*VEGF* and *Tau*) [7,34] or stabilizing (*NEFL*, *HDAC6* and *Add2*) [35,36,37] effects. Although splicing regulation is the main TDP-43 function [4] TDP-43 LOF may also contribute to the neurodegenerative process in ALS and FTLD by different regulatory mechanisms, including the control of mRNA stability and translatability.

When we also investigated the effects of TDP-43 LOF on *GRN* mRNA at the protein level, we observed that an increase in mRNA content was associated with higher levels of intracellular progranulin in both human and murine TDP-43-depleted cells, thus supporting similarity between humans and mice in progranulin regulation. As progranulin is a secreted glycoprotein, whose main functions depend on its extracellular bioavailability [8], we also evaluated its content in conditioned media and we observed that while murine TDP-43 LOF cells showed a parallel increase of extracellular Pgrn levels, no changes of secreted PGRN were detected in the media of TDP-43 LOF human cells. We therefore speculated that the already reported inter-species differences in regulation of *Sortilin* gene expression and splicing mediated by TDP-43 might account for the different effects on extracellular progranulin availability [20,21] (Figure 6).

In murine motoneuronal-like NSC-34 cells, we confirmed that TDP-43 depletion induces *Sort1* splicing changes with an upregulation of the longer *Sort1ex17b* isoform as already described in adult mouse brain and in murine neuroblastoma N2a cells [6,21,38], but without changes of total intracellular Sort1 protein levels, thus suggesting that progranulin uptake via sortilin is not affected by TDP-43 knock-down in mice. In contrast, in human TDP-43-depleted cells, we observed an increase of the main *SORT1∆17b* isoform, as previously described [20], and also of the intracellular SORT1 protein levels, suggesting a possible up-regulation of PGRN uptake by the sortilin receptor that may account for the differences between mice and humans in the availability of extracellular progranulin. Indeed, upon TDP-43 knock-down in murine cells, the increase of extracellular PGRN levels probably depends on a more active secretory pathway while, in human cells, the upregulation of intracellular SORT1 content also favors PGRN uptake, balancing secretion and resulting in unchanged extracellular PGRN levels (Figure 6).

In humans the inclusion of the alternative *SORT1* exon 17b has been described as a less frequent splicing event than in mice, because other RBPs, such as hnRNP L, PTB/nPTB and hnRNP A1/A2, were recently found to participate together with TDP-43 to inhibit human alternative exon 17b splicing [31]. The human truncated SORT1ex17b protein isoform is extracellularly released and is supposed to act as a pathological “decoy receptor”, sequestering the extracellular PGRN [21]. Importantly, the main *SORT1∆ex17b* protein isoform undergoes shedding, a proteolytic cleavage and extracellular release of the large sortilin ectodomain, which still maintains the capacity to bind its ligands [21,39,40]. Sortilin shedding and extracellular release is an important mechanism to regulate the bioavailability of its ligands, including progranulin and BDNF [32], in the extracellular milieu. In our human TDP-43-depleted cells, the cleaved and extracellularly released SORT1 protein slightly decreased, suggesting that both shedding and the SORT1ex17b isoform do not influence PGRN bioavailability, which may rather be influenced by the increased intracellular SORT1 receptor. On the other hand, in murine TDP-43 LOF cells, extracellular sortilin increased by 3- and-5- fold in NSC-34 and N2a cells, respectively, in contrast to the unchanged cellular sortilin content, probably because the longer Sort1ex17b isoform, efficiently generated upon TDP-43 depletion, is suggested to be preferentially cleaved through the shedding mechanism [21]. Therefore, our data suggest that in murine models, conversely to human ones, the extracellularly released sortilin protein may influence progranulin bioavailability rather than the intracellular receptor.

Maintenance of progranulin homeostasis in the nervous system has a pivotal role because decreased progranulin levels may increase neuronal susceptibility to different stressors, which may lead to TDP-43 cleavage and aggregation by autophagy impairment and apoptosis activation [11,12,13,41]. Conversely, progranulin overexpression was shown to be neuroprotective in TDP-43 disease models in vivo, because it is able to rescue neuronal defects and TDP-43 aggregation [25,26,27]. Here we confirm a direct correlation between extracellular progranulin and extracellular cleaved sortilin levels [42] both in humans and mice, although TDP-43 LOF, by differentially regulating *Sortilin* splicing and protein isoform generation, has a different effect on progranulin bioavailability in human versus murine neuronal cells.

Interestingly, the inhibition of PGRN interaction with SORT1 enhances the secreted PGRN levels [18] and the progranulin–sortilin axis has been proposed as a promising therapeutic target to increase PGRN levels in *GRN*-mutated FTLD patients by inhibiting SORT1 expression or its binding affinity to PGRN using small molecules [23]. As the translation of pre-clinical studies in mice to clinical trials is often unsuccessful [43], probably due to inter-species differences, our findings do show different regulatory mechanisms in the progranulin–sortilin axis and suggest that a therapeutic modulation of this pathway may lead to different outcomes in ALS/FTLD mouse models and in patients.

## 4. Materials and Methods 

### 4.1. Cell Culture

Mouse motoneuronal-like NSC-34 cells, a hybrid cell line obtained by fusion of the mouse neuroblastoma cells with motor neuron enriched, embryonic mouse spinal cord cells [44], were maintained in DMEM medium supplemented with 5% fetal bovine serum (FBS, Sigma-Aldrich, St. Louis, MO, USA), 1 mM sodium pyruvate, 100 units/mL penicillin, and 100 μg/mL streptomycin.

Mouse neuroblastoma Neuro-2a cells were cultured in DMEM medium supplemented with 10% FBS, 100 units/mL penicillin, 100 μg/mL streptomycin and 2.5 µg/mL of amphotericin b (Sigma-Aldrich).

Human neuroblastoma M17 and SK-N-BE cells were cultured in RPMI-1640 medium supplemented with 10% FBS, 2 mM L-glutamine, 1 mM sodium pyruvate, 2 g/l glucose, 100 U/mL penicillin and 100 μg/mL streptomycin. Trypsin-EDTA (Sigma-Aldrich) was used to split NSC-34, M17 and SK-N-BE cells.

All neuronal-like cell lines were used in experiments without inducing further neuronal differentiation.

Human embryonic kidney (HEK) 293T cell line was maintained in DMEM supplemented with 10% FBS, 100 U/mL penicillin and 100 μg/mL streptomycin. All media and reagents were from Thermo Scientific if not differently specified.

### 4.2. Plasmid Constructs and Cell Transfection

For loss-of-function experiments, gene silencing was performed using siRNA duplexes for mouse TDP-43 (Sigma) and for human TDP-43 (Ambion, Thermo Fisher Scientific, Waltham, MA, USA), as reported in [7,20]. Stealth RNAi Negative Control Low GC (Life Technologies) was used as control siRNA. For the experiments, 1.5 × 10^5^ cells/well were plated in 6-well plates and silenced in a double round using Lipofectamine 2000 (Thermo Fisher Scientific). NSC-34 and Neuro-2a cells were harvested after 96 h of gene silencing with the siRNA duplex (40 nM), while M17 and SK-N-BE cells were silenced with siRNA duplex (80 nM) for 72 h. For quantification of secreted progranulin levels, the culture medium was replaced by Optimem (Thermo Fisher Scientific) 16 h before harvesting cells. 

For the gain-of-function experiments, 2 × 10^5^ NSC-34 or M17 cells/well were seeded in 6-well plates for Western blotting and on glass coverslips for the immunostaining assay and transiently transfected with 3 μg of pGFPC2 empty vector (AddGene, Watertown, MA, USA), pGFPC2-hTDP-43, pGFPC2-hTDP-35 or pGFPC2-hTDP-25 (all a kind gift of Dr. E. Buratti, ICGEB, Trieste). After a 48 h transfection, cells for immunostaining were fixed in 4% paraformaldehyde in phosphate buffered saline (PBS, pH 7.4, Santa Cruz Biotechnology, Dallas, TX, USA) for 20 min at room temperature (RT). Differently, for Western blot analyses, cells were harvested, used to generate lysate and processed as described below.

For UV-CLIP assays, 1.5 × 10^6^ HEK293T cells were seeded in a 100 mm dish, transiently transfected with 6 μg of pFlag-CMV2-hTDP-43 (a kind gift of Dr. E. Buratti, ICGEB, Trieste, Italy), harvested 24 h after transfection and used to generate lysate for protein extraction, as described below.

### 4.3. Western Blot Assay

Protein lysates were obtained by homogenizing cells in lysis buffer (150 mM NaCl, 20 mM Tris–HCl pH 7.4, 1% Triton X-100, protease inhibitor cocktail (Roche Italia, Monza, Italy). Protein lysates were incubated for 15 min on ice and then quantified by BCA protein assay (Thermo Fisher Scientific). Protein samples (25 μg) were resolved by SDS-PAGE on 10% NuPAGE Bis-Tris pre-cast polyacrylamide gels (Thermo Fisher Scientific) and transferred to nitrocellulose membranes (pre-cast iBlot Transfer Stack, Thermo Fisher Scientific).

For the quantification of secreted progranulin, centrifugation of the conditioned media at 4000× *g* for 5 min was performed to remove debris and dead cells and the supernatants were collected. One-fifth of the total medium was precipitated overnight at −20 °C in four volumes of cold acetone. The day after, media were centrifuged at 15,000× *g* at 4 °C for 30 min and pellets were resuspended in SDS-loading buffer and resolved by SDS-PAGE as described above.

Specific primary antibodies (reported in Supplementary Table S1) diluted in blocking solution (5% milk in TBS (Santa Cruz Biotechnology) with 0.1% Tween-20 (Sigma-Aldrich)) were used to perform immunoblots and chemiluminescence detection was obtained by the Novex ECL kit (Thermo Fisher Scientific). Quantity One software (Biorad, Hercules, CA, USA) was used for densitometric analyses. For quantification of secreted progranulin, the densitometric raw data were normalized on the total protein amount of the corresponding cellular lysate.

### 4.4. ELISA Assay

The mouse progranulin ELISA Kit (Adipogen_AG-45A-0019Y, San Diego, CA, USA) was used to quantify the Pgrn protein content in the conditioned media of NSC-34 cells upon TDP-43 knockdown and a 1/25 of the total conditioned medium was used for quantification according to the manufacturer’s instruction. 

### 4.5. RNA Isolation, Reverse Transcription (RT) and Real Time PCR

Total RNA was obtained using TriZol reagent (Thermo Fisher Scientific) following the manufacturer’s instructions. Total RNA from NSC-34/N2a (1 μg) and M17/SK-N-BE (3 μg) cells was treated with 1 U DNaseI (Roche) for 20 min at 37 °C and retro-transcribed using oligodT and 1 U SuperScript II-RT (Thermo Fisher Scientific).

Real time PCR was performed with SYBR Green PCR Master mix (Applied Biosystems, Thermo Fisher Scientific) on QuantStudio 12 K Flex (Applied Biosystems) and the primer sequences used for human and mouse *Grn*, total *Sort1, Sort1∆ex17b* and *Sort1ex17b* isoforms, and *RPL10a* genes are listed in Supplementary Table S2. Threshold cycles (*C*t) of the housekeeping *RPL10a* gene were used to normalize the *C*t value (Δ*C*t) for each tested gene and every experimental sample was referred to the mean Δ*C*t of the controls (ΔΔ*C*t). Fold change values were expressed as 2^−ΔΔ*C*t^.

### 4.6. Immunofluorescence and Image Acquisition

Cells were fixed as described above were then washed twice in 1× PBS for 10 min and permeabilized in 0.3% Triton X-100 (Sigma) in 1× PBS (Gibco, Thermo Fisher Scientific) for 5 min at RT. After blocking buffer (5% of Normal Goat Serum (Gibco) in PBS,) for 30 min at RT, incubation with anti-GFP antibody (1:300; Roche) was performed for 90 min at 37 °C. Anti-mouse IgG Alexa Fluor 488 (1:500, Thermo Fisher Scientific) was used as secondary antibody and coverslips were mounted onto glass slides using Prolong Gold antifade reagent with DAPI (Thermo Fisher Scientific). A confocal inverted microscope (Nikon Eclipse C1) was used to acquire Z-stacks images (0.2 μm) with a 60× magnification. 

### 4.7. Nucleotide Sequence Alignment and in silico Analysis 

The nucleotide sequence alignment of human (NM_002087.3) and murine (NM_008175.5) *GRN* 3′UTR was performed by the online pairwise sequence alignment EMBOSS Needle software. The online RBPmap software (http://rbpmap.technion.ac.il [45]) was used to predict the putative TDP-43 binding region in the human *GRN* 3′UTR. A stringent cut-off (≥5) was set for the Z-score value. 

### 4.8. UV-Cross-Linking and Immunoprecipitation (UV-CLIP)

The 3′UTR region sequence of human *GRN* (322 pb) was amplified by RT-PCR from SK-N-BE total RNA using the primers listed in Supplementary Table S2, cloned into the TOPO TA vector (Thermo Fisher Scientific) downstream of the T7 promoter and linearized (0.5 μg) by HindIII restriction enzyme (30 U) for in vitro transcription. The mouse *Grn* 3′UTR probe [7] was used as a positive control. UV-crosslinking was performed using HEK293T protein lysates (200 μg) transfected with human Flag-TDP-43 and ^32^P-radiolabeled riboprobes, as previously described [46]. Immunoprecipitation was conducted on UV-crosslinked samples using the anti-FLAG (2 μg; Sigma-Aldrich) or the anti-IgG antibodies (2 μg, SantaCruz Biotechnology) (Supplementary Table S1) and protein G Dynabeads (Thermo Fisher Scientific). Immunocomplexes were washed several times in PBS with 0.02% Tween-20, run on a 10% SDS-PAGE gel and analyzed by autoradiography.

### 4.9. Statistical Analyses

Statistical analyses were performed using two-tailed unpaired student’s *t* test or one-way ANOVA, followed by appropriate post-hoc tests to compare two or multiple groups, using the GraphPad PRISM 5 software package. Data were presented as mean ± standard error of mean (s.e.m.) of at least three independent experiments and significance value was defined as * *p* < 0.05, ** *p* < 0.01, *** *p* < 0.001.

## 5. Conclusions

Our results show that the progranulin–sortilin axis is differently affected by TDP-43 loss-of-function in mice and humans because although the TDP-43-dependent regulation of *Progranulin* mRNA is conserved, the different control of sortilin gene expression, splicing, protein cleavage and extracellular release strongly influences the bioavailability of extracellular progranulin in the two species. As the progranulin–sortilin axis has been proposed as a novel therapeutic target in ALS/FTLD, inter-species differences in regulatory mechanisms associated to the TDP-43 protein should be carefully considered when translating preclinical data obtained in mouse disease models to patients.

## Supplementary Materials

Supplementary materials can be found at https://www.mdpi.com/1422-0067/20/23/5866/s1.

## Abbreviations

ALS	amyotrophic lateral sclerosis
FTLD	frontotemporal lobar dementia
RBP	RNA-binding protein
TDP-43	TAR DNA binding protein 43
PGRN	progranulin
SORT1	sortilin 1
LOF	loss of function
GOF	gain of function
