# Supplementary material for: Inter-Species Differences in Regulation of the Progranulin–Sortilin Axis in TDP-43 Cell Models of Neurodegeneration
Source: Int J Mol Sci. 2019 Nov 22;20(23):5866. doi: 10.3390/ijms20235866 (PMC6928728; doi:10.3390/ijms20235866)
Supplement: Supplementary file 1 [file ijms-20-05866-s001.pdf]

SUPPLEMENTARY MATERIALS

Supplementary Figure 1

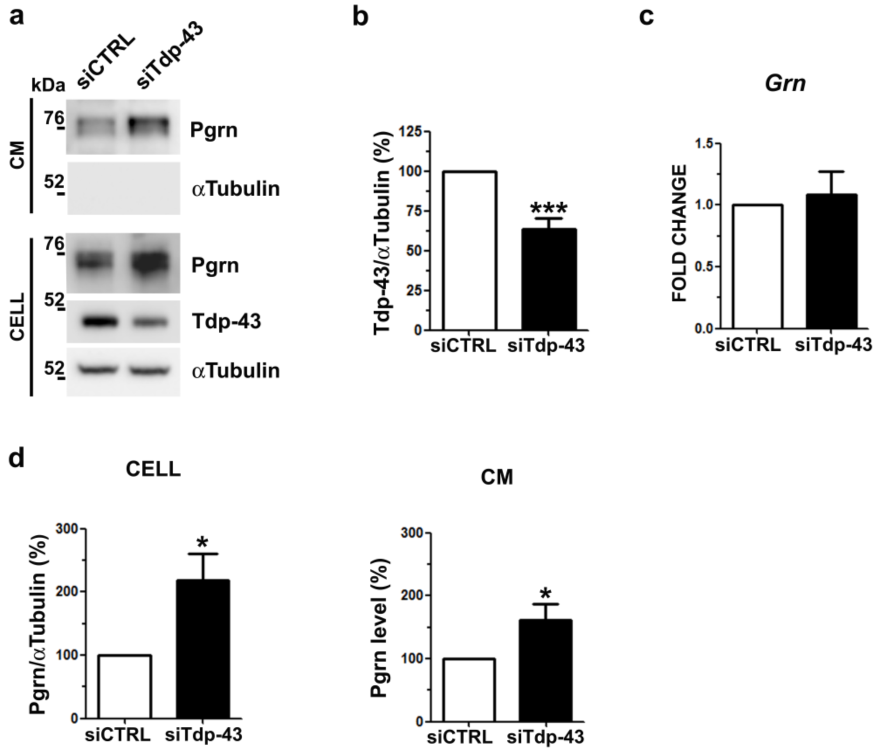

Analysis of intracellular and secreted Pgrn levels upon Tdp-43 LOF in murine neuroblastoma N2a cells.

a) Representative WB images of Pgrn levels in cell lysates (CELL) and in conditioned media (CM) upon Tdp-43 depletion in murine N2a cells. Tdp-43 immunoblot was reported to show gene silencing efficiency, α-Tubulin was used for data normalization and as negative control in conditioned media. **b**) Densitometric and statistical analyses of Tdp-43 protein levels shown in (**a**). **c**) Real time PCR of *Grn* gene expression upon Tdp-43 depletion. **d**) Densitometric and statistical analyses of intracellular (CELL) and secreted (CM) Pgrn levels in Tdp-43 depleted N2a cells shown in (**a**) (mean±s.e.m.; n=4 independent experiments; Two-tailed Unpaired *t* test; * p< 0.05, *** p<0.001).)

Supplementary Figure 2

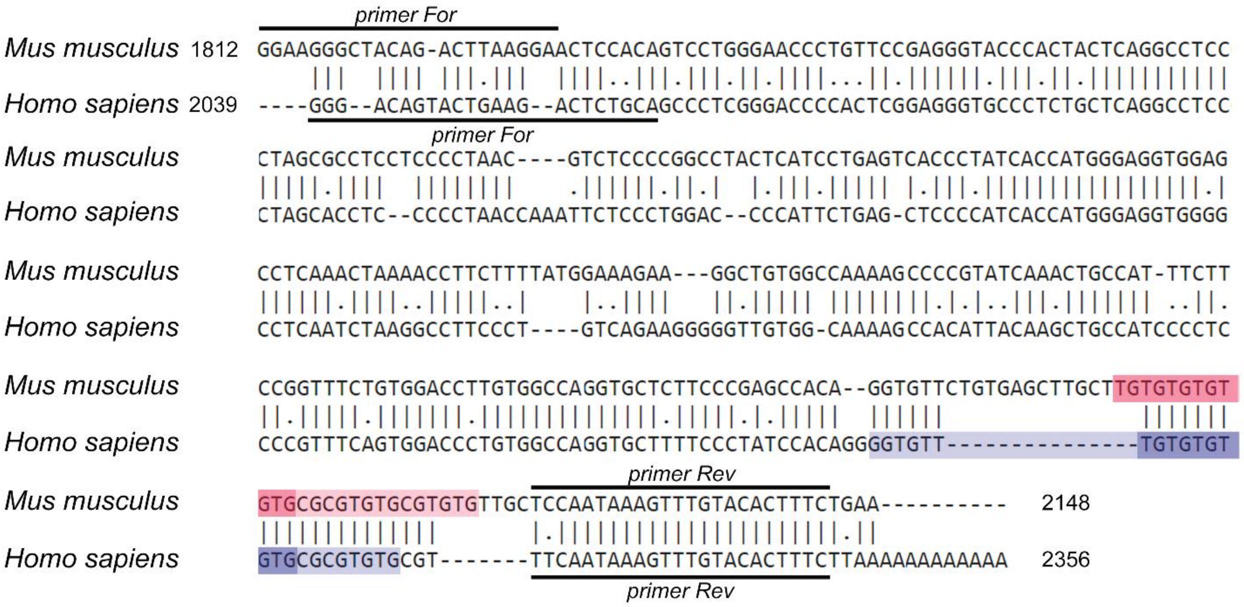

Human and murine *GRN* 3'UTR sequence alignment and the putative TDP-43 binding region. The human (NM_002087.3) and murine (NM_008175.5) *GRN* 3'UTR sequence alignment performed by the pairwise sequence alignment EMBOSS Needle software. Red boxes were used to highlight the putative binding site for TDP-43, containing the $(TG)_6$ motif (dark red box), in the murine sequence. Blue boxes indicate the TDP-43 binding region in the human *GRN* 3'UTR sequence predicted by RBPmap software setting a stringent Z-score ≥5. The $(TG)_5$ motif is highlighted (dark blue box). The primer sequences used for cloning of the murine and human 3'UTR sequences are indicated.

Supplementary Figure 3

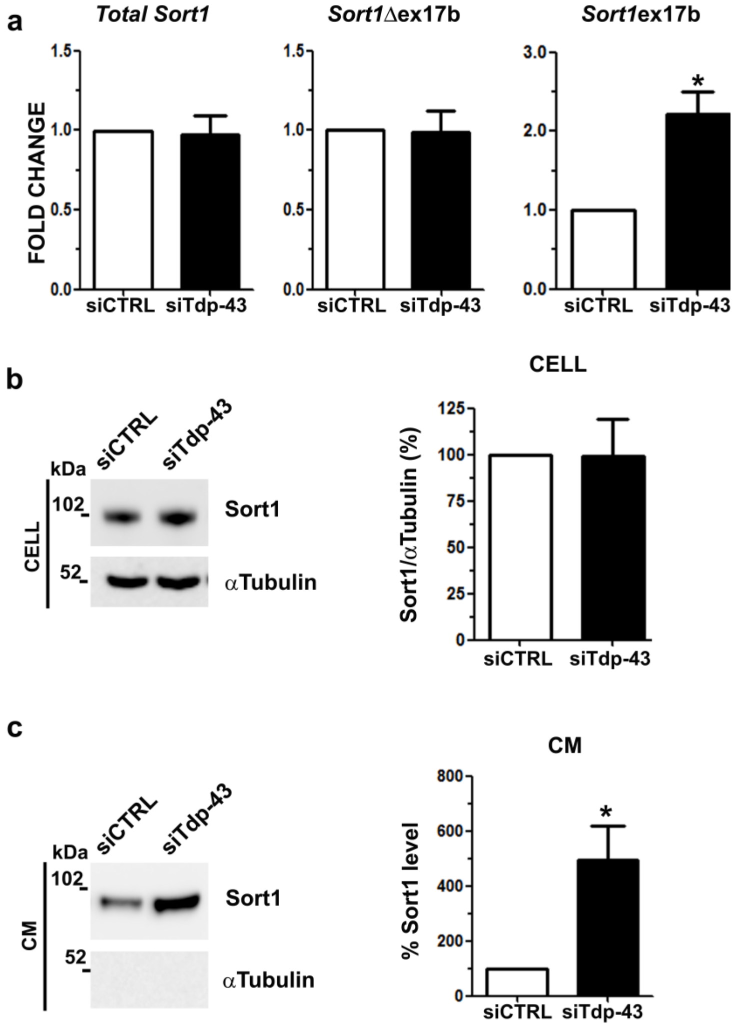

Sortilin1 splicing and protein levels upon Tdp-43 *LOF* in murine neuroblastoma N2a cells.

a) Real time PCR of total and splicing isoforms of *Sort1* in Tdp-43 knocked-down murine neuroblastoma N2a cells shown in Supplementary Figure 1. **b)** Representative WB images and densitometric analysis of total intracellular (CELL) Sort1 protein levels upon Tdp-43 knock-down in N2a cells. α-Tubulin was used for data normalization. **c)** Representative WB and densitometry data of Sort1 levels in the conditioned media (CM) (mean±s.e.m.; n=4 independent experiments; Two-tailed Unpaired *t* test; * $p < 0.05$).

Supplementary Figure 4

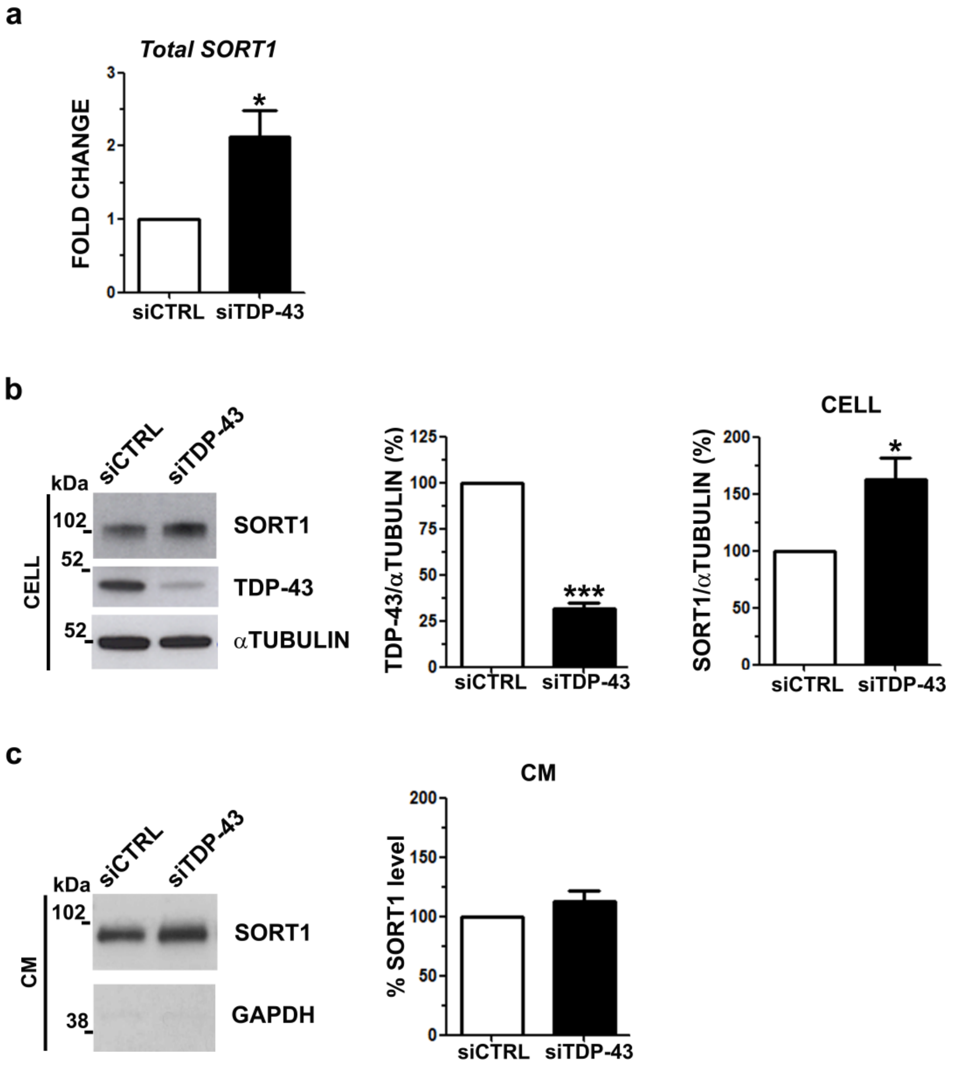

Sortilin1 splicing and protein levels upon TDP-43 *LOF* in human neuroblastoma SK-N-BE cells.

a) Real time PCR of total *SORT1* in condition of TDP-43 gene silencing in human neuroblastoma SK-N-BE cells (mean±s.e.m.; n=4 independent experiments; Two-tailed Unaired *t* test; * p< 0.05). **b)** Representative WB images and densitometric analysis of total SORT1 protein levels upon TDP-43 knock-down in SK-N-BE cells. TDP-43 immunoblot was performed to assess gene silencing efficiency and α-Tubulin was used for data normalization (mean±s.e.m.; n=4 independent experiments; Two-tailed Unpaired *t* test; * p< 0.05; *** p< 0.001). **c)** Representative WB and densitometry data of SORT1 levels in the conditioned media (CM). GAPDH immunoblot was performed as negative control(mean±s.e.m.; n=3 independent experiments; Two-tailed Unpaired *t* test).

Supplementary Table S1. List of the primary antibodies used for immunofluorescence (IF) and Western blot (WB) assays.

Antibody	Source	Assay
GFP (1:2000)	Roche_11814460001	IF/WB
PGRN (mouse) (1:1000)	R&D Systems_AF2557	WB
PGRN (human) (1:300)	Invitrogen_40-3400	WB
TDP-43 (1:1000)	Protein Tech_10782-2-AP	WB
SORT1 (1:300)	R&D Systems_MAB3154	WB
α Tubulin (1:500)	Sigma-Adrich_T6199	WB
POLDIP3 (1:500)	Cell Signaling_5439	WB
FLAG (1:1000)	F3165 (Sigma-Aldrich)	IP
goat anti-mouse IgG	sc-2025 (Santa Cruz)	IP

Supplementary Table S2. List of primers sequences used for real time PCR.

Gene	Foward primer	Reverse primer	Ref
murine GRN	CCAACTACAGCTGCTGTAAC	CTCGTTATTCTAGGCCATGTG	[7]
murine SORT1 (Total)	CGTGTTCCCTGGAGGACTTCCT	TTCAGGCTGCTCCACGCACT	[21]
murine SORT1ex17b	AAATCCCAGGAGACAAATGC	GAGCTGGATTCTGGGACAAG	[21]
murine SORT1Δex17b	CCCCACAAAGCAGAATTCCAAGTC	TGACAAGCATCAGTCCCACGAT	[21]
murine RPL10a	GAAGAAGGTGCTGTGTCTGGC	TCGGTCATCTTCACGTGGC	[7]
human GRN	CAGCTACAGCTGCTGCCGTC	CTCAGTGTTGTGGGCCATTTG	-
human SORT1 (Total)	GGCCTGTGGGTGTCCAAGAA	GCAGGAGCCATTTGCATAGGTT	[21]
human SORT1ex17b	AATCCAGCTCTGCCTCCTCT	TCCCACGATGGCCAGGATAA	[21]
human SORT1Δex17b	TGGGGTAAATCCAGTTCGAG	GACTTGGAATTCTGTTTTTCCGGAC	[21]
human RPL10a	GAAGAAGGTGTTATGTCTGG	TCTGTCATCTTCACGTGAC	[20]
